# The Adult Phenylketonuria (PKU) Gut Microbiome

**DOI:** 10.3390/microorganisms9030530

**Published:** 2021-03-04

**Authors:** Viviana J. Mancilla, Allison E. Mann, Yan Zhang, Michael S. Allen

**Affiliations:** 1 Department of Microbiology, Immunology, and Genetics, Graduate School of Biomedical Sciences, University of North Texas Health Science Center, Fort Worth, TX 76107, USA; amann3@clemson.edu (A.E.M.); yan.zhang@unthsc.edu (Y.Z.); 2 Department of Biological Sciences, Clemson University, Clemson, SC 29634, USA

**Keywords:** microbiome, phenylketonuria (PKU), 16S rRNA, gastrointestinal microbiota, metabolism, diet, diversity

## Abstract

Phenylketonuria (PKU) is an inborn error of phenylalanine metabolism primarily treated through a phenylalanine-restrictive diet that is frequently supplemented with an amino acid formula to maintain proper nutrition. Little is known of the effects of these dietary interventions on the gut microbiome of PKU patients, particularly in adults. In this study, we sequenced the V4 region of the 16S rRNA gene from stool samples collected from adults with PKU (*n* = 11) and non-PKU controls (*n* = 21). Gut bacterial communities were characterized through measurements of diversity and taxa abundance. Additionally, metabolic imputation was performed based on detected bacteria. Gut community diversity was lower in PKU individuals, though this effect was only statistically suggestive. A total of 65 genera across 5 phyla were statistically differentially abundant between PKU and control samples (*p* < 0.001). Additionally, we identified six metabolic pathways that differed between groups (*p* < 0.05), with four enriched in PKU samples and two in controls. While the child PKU gut microbiome has been previously investigated, this is the first study to explore the gut microbiome of adult PKU patients. We find that microbial diversity in PKU children differs from PKU adults and highlights the need for further studies to understand the effects of dietary restrictions.

## 1. Introduction

Phenylketonuria (PKU, OMIM 261600) is a genetic disorder characterized by dysfunctional metabolism of the essential amino acid phenylalanine (Phe) and occurs in approximately 1 in 10,000 births per year worldwide [1]. Disrupted phenylalanine metabolism can be caused by an autosomal recessive mutation in the PAH gene, resulting in a dysfunctional phenylalanine hydroxylase (PAH), or lack of its cofactor tetrahydrobiopterin (BH4) [2]. When dietary Phe is consumed in a healthy system, it is absorbed in the gut and transported through the blood to the liver where it is metabolized to tyrosine by PAH. In PKU patients, a lack of functional PAH or BH4 leads to the accumulation of Phe and its derivatives in the blood. If left untreated, accumulation can reach neurotoxic levels, and result in an increased incidence of developmental problems, including intellectual disabilities, growth failure, and seizures. 

Worldwide, newborns undergo routine screenings for several disorders, including PKU [3]. Upon detection of above-average concentrations of phenylalanine in the blood, an infant will proceed to further testing in order to determine a diagnosis for PKU. Typically, Phe concentrations range from 1.3 to 2.0 mg/dL in the blood, but in classic PKU, blood Phe concentrations can exceed 20 mg/dL [4]. Immediately after diagnosis, the infant is prescribed a phenylalanine-restricted diet supplemented with an amino acid formula. Although this is currently the most effective and only universal treatment for PKU, Phe is an essential amino acid found in most complex proteins and whole foods. Therefore, the PKU diet can be low in protein, fiber, and other important nutritional sources; thus leading to difficulty in patient compliance and nutritional deficiencies [4,5]. Nutritional deficiencies include, but are not limited to, vitamin B12, iron, and long-chain fatty acids [6,7,8].

Active research and developments aim to provide patients with alternative treatment options for PKU. Of note, a recent study found that inhibition of SLC6A19, a neutral amino acid transporter, led to decreased Phe in plasma and increased urinary excretion of Phe [9]. Enzyme replacement therapy aims to reduce Phe through the use of enzymes capable of metabolizing Phe, for example, rAvPAL-PEG (PEGylated recombinant phenylalanine ammonia lyase) which transforms phenylalanine to ammonia and trans-cinnamic acid [10]. Additionally, probiotics have been engineered to express phenylalanine-metabolizing enzymes upon oral administration by the host [11,12]. Administration of PAL as a therapeutic option has previously proved to be difficult due to issues with stabilization and high cost; however, the probiotic provides protection from stomach acids, allowing for the enzymes to be expressed later in the digestive tract where it can become metabolically active, ultimately reducing Phe in the host [13].

The gut microbiome is a complex collection of microbes housed primarily in the colon and has been found to influence the physiology of distal organs through association with diseases such as type 2 diabetes, inflammatory bowel disease (IBD), and asthma [14,15,16]. Gut microbiome activity has been suggested to alter blood–brain barrier permeability and has also been linked to psychiatric disorders including autism, schizophrenia, and anxiety [17,18,19,20]. 

The composition of the gut microbiome is affected by many factors including medications, birth route (vaginal or cesarean), and host genetics, but is primarily shaped by diet [21,22]. Previous studies have investigated the impact of the PKU treatment diet and Phe restriction on children and have found notable differences in the gut microbial community as compared to non-PKU controls [23,24]. Though these studies document the impact of PKU treatment in children, little is known about the impact of the PKU diet in adults. While the development of a probiotic for the treatment of PKU is a promising approach, little is known about the baseline community of the gut microbiome of PKU patients over the entire lifespan; the composition of which has direct implications on the success or impact of any therapeutic probiotic that becomes available [25]. In this study, we examine the gut microbiome of 32 adults with and without PKU using a 16S rRNA gene fragment metabarcoding approach. 

As the PKU diet is extremely restrictive and characteristically different than that of the general population, we expected the composition of the PKU-treated gut microbiome to be markedly different from the control group in both diversity and taxonomic composition, with subsequent changes in metabolic capacity and activity. To our knowledge, this is the first study to catalog the gut microbiome of adults with PKU. Characterization of the PKU adult gut microbiome may prove beneficial to the development of new therapeutics or improvement of treatment options currently available.

## 2. Materials and Methods

### 2.1. Sample Collection

A total of 32 (11 PKU, 21 non-PKU controls) stool samples were collected by participants using a Puritan HydraFlock^®^ swab in DNA/RNA Shield (Puritan Medical Products, Guilford, ME, USA), returned to laboratory personnel, and stored at –20 °C until ready for DNA isolation and quantification. 

The average age of control subjects was 29 years (±3.07). Both control and PKU samples were collected from approximately equal numbers of males and females. The average age of PKU subjects was 33 years (±1.98). Control samples were collected from 11 females and 10 males, PKU samples from 4 females, 6 males, and 1 N/R (sex not reported by one participant) (Table 1). The study was approved by the North Texas Regional Institutional Review Board (IRB Protocol#2018-114, January 2018 #2018-193, August 2019).

### 2.2. DNA Isolation and Quantification

Fecal samples were thawed on ice and DNA was extracted using the Kingfisher MagMAX Microbiome Ultra Nucleic Acid Isolation Kit (Thermo Fisher Scientific Inc., Waltham, MA, USA) following the manufacturer’s protocol. The final elution volume was adjusted to 50 μL. The extracted DNA was quantified using a NanoDrop 1000 Spectrophotometer (Thermo Fisher Scientific Inc., Waltham, MA, USA) and stored at –20 °C.

### 2.3. 16S rRNA Gene Amplification

The V4 hypervariable region of the 16S rRNA gene was amplified in duplicate from the previously extracted DNA utilizing illumv4_515F (5′–GTGCCAGCMGCCGCGGTAA–3′) and illumv4_806R (5′–GGACTACHVGGGTWTCTAAT–3′) primers with Illumina sequencing adaptors [26]. PCR reactions were prepared in 25 μL volumes consisting of 2.5 μL 10 × Accuprime™ PCR Buffer II (Invitrogen, Carlsbad, CA, USA), 0.5 μL of forward and reverse primer (10 μM), 0.1 μL of Accuprime™ Taq DNA Polymerase High Fidelity (5U/μL), 1 μL of template DNA (10–100 ng), and 16.9 μL of molecular grade water. Negative and positive controls were produced along with every master mix containing either 1 μL of *Escherichia coli* genomic DNA or 1 μL of molecular grade water, as appropriate. Thermocycler conditions included an initial annealing at 94 °C for 2 min followed by 30 cycles of 94 °C for 30 s, 55 °C for 40 s, 68 °C for 40 s, and a final extension at 68 °C for 5 min. PCR products were visualized on a 1% agarose gel. Bands were observed in all positive PCR controls and absent for all negative controls.

### 2.4. Library Preparation and Sequencing

Duplicate PCR products were pooled into a single tube and purified using Agencourt^®^ AMPure^®^ XP magnetic beads (Beckman Coulter, Brea, CA, USA) following the manufacturer’s instruction. Purified samples were then dual indexed using the Nextera XT assay to allow for multiplexing. The 50 μL PCR recipe and thermocycler protocol were as follows per sample: 5 μL 10 × Accuprime™ PCR Buffer II, 5 μL of Nextera XT Index Primer 1, 5 μL of Nextera XT Index Primer 2, 0.2 μL of Accuprime™ Taq DNA Polymerase High Fidelity, 5 μL of DNA (~10 pm DNA), and 29.8 μL of molecular grade water; initial denaturation at 94 °C for 3 min, 8 cycles of 94 °C for 30 s, 55 °C for 30 s, and a final extension at 68 °C for 5 min. Labeled products were purified as previously described by AMPure^®^ XP magnetic beads and quantified using the Qubit^®^ 2.0 fluorometer (Invitrogen, Carlsbad, CA, USA). Following quantification, libraries were pooled at equimolar concentrations and then diluted and denatured for a final concentration of 10 pM. The product was loaded into the MiSeq Reagent Kit v2 cartridge (Illumina Inc., San Diego, CA, USA) and sequenced on the Illumina MiSeq^®^ instrument, along with an internal control sample consisting of 5% PhiX DNA, for paired-end high-throughput sequencing at 500 cycles.

### 2.5. Sequencing Data Analysis 

Primers were trimmed from raw reads using Cutadapt v1.16 [27]. Trimmed reads were quality filtered, merged, checked for chimeras, and clustered into amplicon sequencing variants (ASVs) using the DADA2 pipeline [28]. Finally, taxonomy was assigned to processed sequences using VSEARCH v2.8.1 and the EzBiocloud database as a reference. Data analysis was primarily performed within the R version 3.5.0 environment [29,30]. Alpha diversity was calculated using phyloseq v1.30.0 [31]. Beta diversity was calculated using PhILR transformed data and the Vegan v2.5−5 library [32,33]. Analysis of variance was determined via permutational multivariate analysis of variance (PERMANOVA) [34]. Differential abundance of specific bacteria or phylogenetic clades between PKU positive and control samples was calculated with DESeq2 and Phylofactor [35,36]. To predict differences in the metabolic potential between the PKU gut microbiome compared to the control gut microbiome, we utilized the PICRUSt2 (Phylogenetic Investigation of Communities by Reconstruction of Unobserved States) pipeline [37]. Visualization of metagenome imputation was then completed with STAMP (Statistical Analysis of Metagenomic Profiles) [38]. The script for all read processing, analyses, and visualization can be accessed at https://github.com/vivmancilla/PKU_adults (accessed on 25 February 2021).

## 3. Results

### 3.1. Gut Microbial Composition of PKU Patients Diverges from Control Individuals

**Figure 1:**
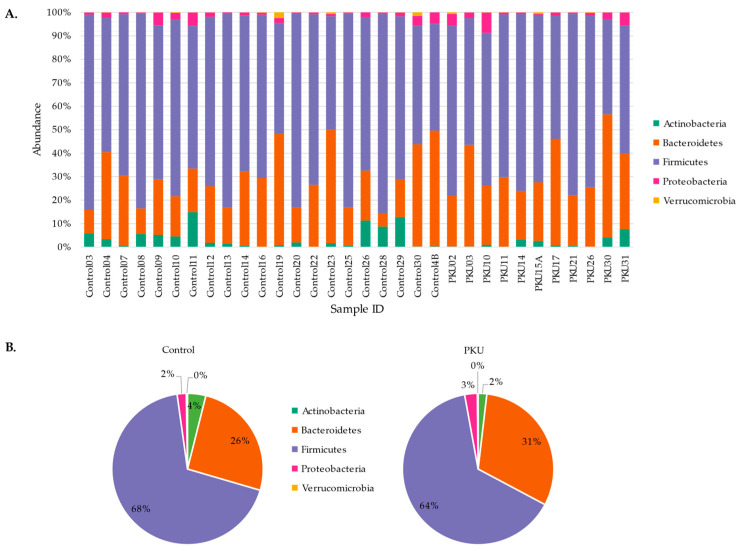
Phylum-level comparison between PKU and control samples. Stacked histogram (**A**) and pie graph (**B**) representing the individual and average abundance of phyla (> 1% relative abundance) in PKU and control samples.

Five bacterial phyla were detected in both the PKU and control groups (based on a > 1% abundance). Firmicutes was the predominant bacterial phyla in both PKU (63.55 ± 12.88%) and control cohorts (68.15 ± 12.86%) (Figure 1). Bacteroidetes was the second most abundant population in both PKU (30.55 ± 9.86%) and control cohorts (25.57 ± 13.19%) followed by Actinobacteria, Proteobacteria, and Verrucomicrobia (<10%). 

To identify specific groups or clades of bacteria that were predictive of PKU status, we ran two separate analyses, DESeq2 and Phylofactor [35,36]. While the motivation for running these analyses are the same, DESeq (ASV-based pairwise comparison) identifies broader differences in taxa abundance between PKU and control samples while Phylofactor searches for potential bioindicators of PKU.

**Figure 2:**
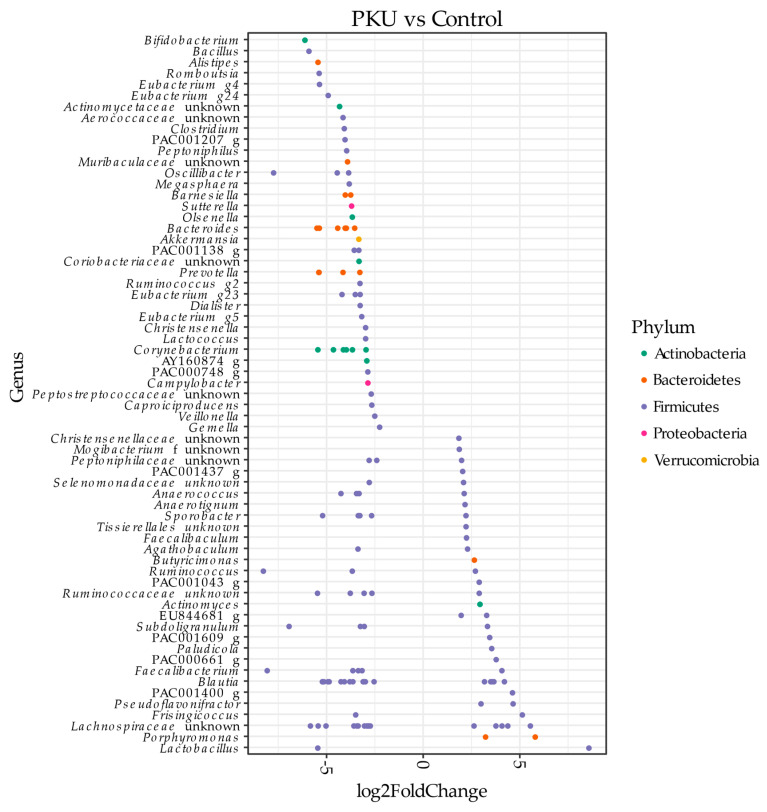
Pairwise comparison (DESeq2 analysis). Differentially abundant amplicon sequencing variants (ASVs) (*p* < 0.001) in PKU patients and non-PKU control counterpart samples are shown. ASVs were assigned to the genus (y-axis) and phylum level (colors). Negative “log2FoldChange” values (x-axis) indicate higher abundance in PKU samples and positive values indicate higher abundances in control samples.

DESeq2 identified 65 differentially abundant genera (*p* < 0.001) in PKU compared to control samples (Figure 2). Genera enriched in PKU samples include *Bifidobacterium, Bacillus, Alistipes, Clostridium, Akkermansia,* and *Bacteroides*. Alternatively, genera that decreased in PKU samples include *Lactobacillus, Porphyromonas, Frisingicoccus, Blautia,* and *Faecalibacterium*. 

**Figure 3:**
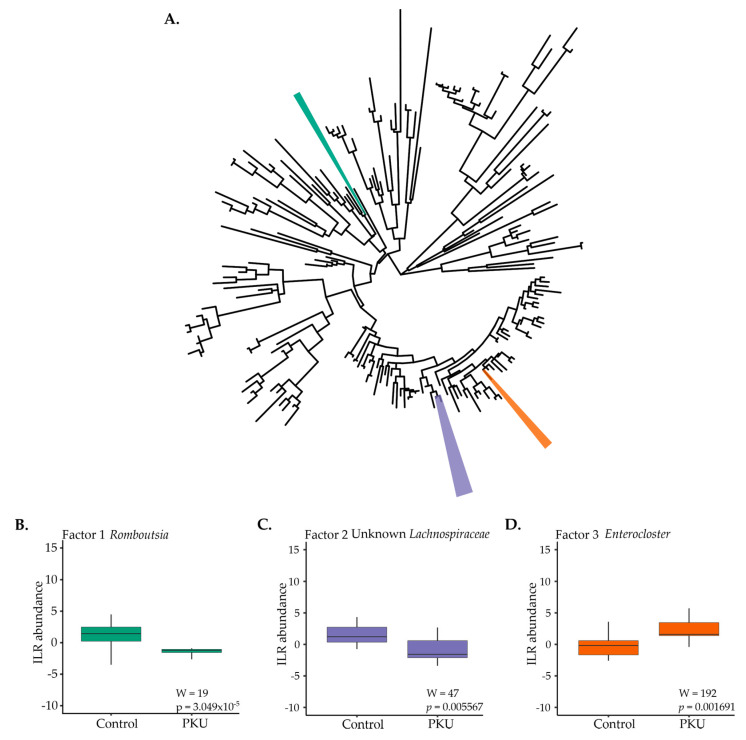
Phylofactorization of all bacterial ASVs present in PKU and control fecal samples (**A**). Three factors were identified as the most influential members differentiating PKU and control samples at the genus level: (**B**) *Romboutsia* (green), (**C**) two unknown Lachnospiraceae (purple), and (**D**) *Enterocloster* (orange).

Phylofactor analysis, or ”phylofactorization”, of all bacterial ASVs present in the PKU and control dataset identified three phylogenetic factors influencing microbial community composition (Figure 3) [35]. F-statistics identified “bioindicator” clades between conditions (Figure 3A). Clades of interest for PKU in comparison to control samples included: *Romboutsia* (green; *W* = 19, *p* = 3.049 × 10^−5^) was lower in PKU samples (Figure 3B), Lachnospiraceae (purple; *W* = 47, *p* = 0.005567) was lower in PKU samples (Figure 3C), and *Enterocloster* (formerly classified as *Clostridium* in EZBioCloud database [39]) (orange; *W* = 192, *p* = 0.001691) was higher in PKU samples compared to control samples (Figure 3D). 

### 3.2. Comparison of Microbial Diversity of PKU and Control Samples

**Figure 4:**
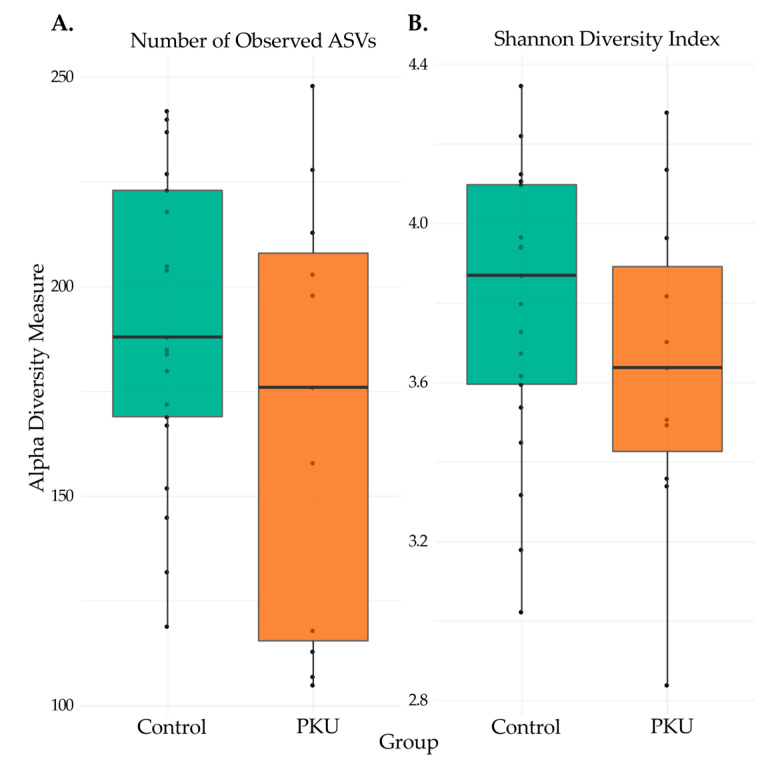
Alpha diversity for PKU and control samples. Statistical significance of the (**A**) number of observed ASVs and (**B**) Shannon diversity index from PKU and control samples were calculated and compared with the Wilcoxon rank-sum test. Median scores are represented with a horizontal line, the box outer limits represent the first to third quartiles, the whiskers indicate the range of measurements for each group, and each point denotes a sample. Observed ASVs (Wilcoxon: *W* = 143, *p* = 0.28) and Shannon index (Wilcoxon: *W* = 139, *p* = 0.37).

Alpha diversity, the variation of microbes within sample groups, was estimated with both ASV richness (Figure 4A) and Shannon diversity index (Figure 4B). Richness was measured by a count of distinguishable ASVs in each sample group. Shannon diversity index accounts for the richness and diversity (distribution of ASV abundance) of the sample groups. Adult PKU patients had lower, but not statistically significant, differences in microbial diversity as measured by both the number of observed ASVs (Wilcoxon: *W* = 143, *p* = 0.28) and Shannon index (Wilcoxon: *W* = 139, *p* = 0.37). 

**Figure 5:**
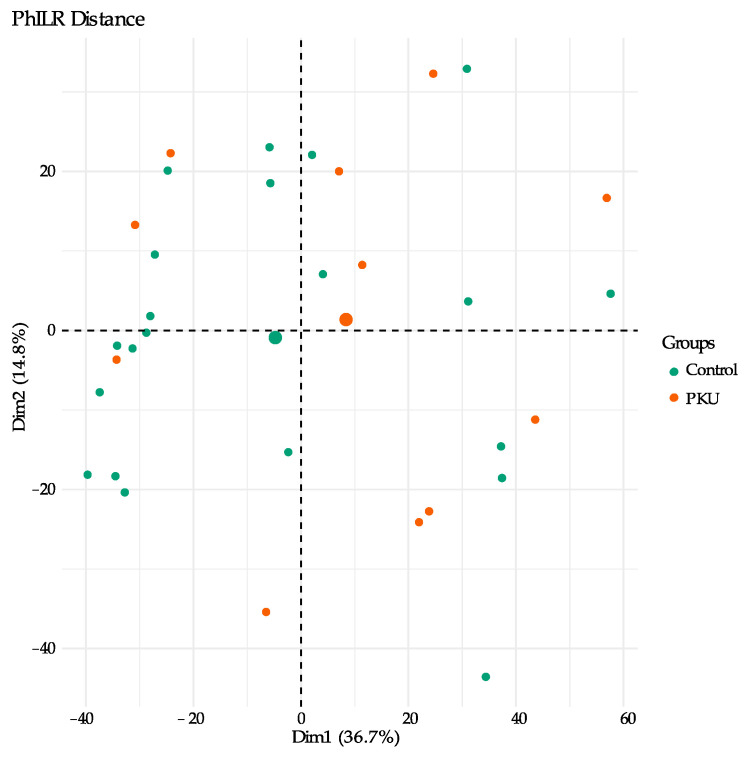
Genus-level beta diversity comparison of the gut microbiota compositions based on principal component analysis (PCA) of PhILR distances for PKU (orange) and control (green) samples. Each point represents a sample, and the condition is denoted by its color; larger circles represent the mean coordinates for each group.

Beta diversity, the variation of microbial communities between sample groups, was estimated by way of principal component analysis (PCA) of phylogenetic isometric log-ratio (PhILR) transformed distances for PKU and control samples (Figure 5). PhILR utilizes a phylogenetic approach to transform microbiome data in order to produce coordinates which can then be used to produce PCA plots [32]. In PCA plots, individual points represent compressed data found for individual samples and located based on dissimilarity of microbial communities. Analysis of the variance of the “data cloud”of each sample group through PERMANOVA determined that the differences in impact of samples based on group (PKU vs. control) were statistically significant (R^2^ = 0.06, *p* = 0.02), though this only explained about 6% of the total diversity [34].

### 3.3. Metagenome Imputation Analysis

**Figure 6:**
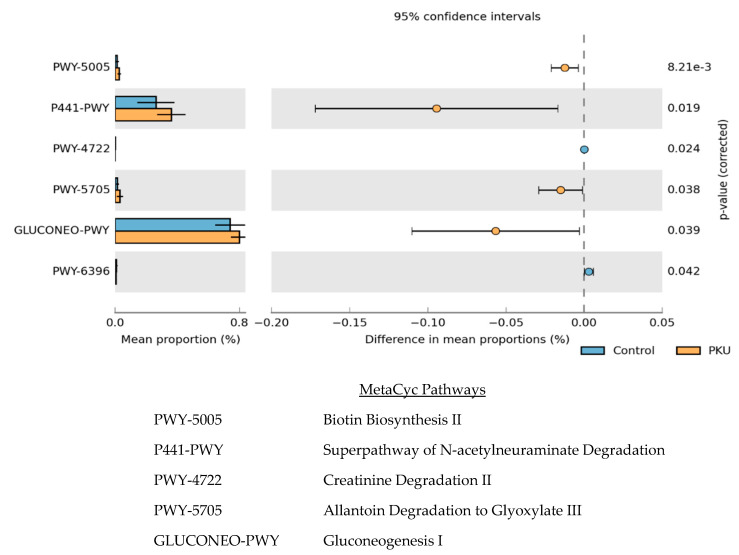
The relative abundance of predicted microbial genes related to metabolism in control (blue) and PKU (orange) samples was based on Welch’s t-test (*p* ≤ 0.05). The colored bars represent 95% confidence intervals calculated using Welch’s inverted method. The colored circles represent the difference in mean proportions between PKU and control samples.

To predict the effect of PKU on the metabolic function of the gut microbial community compared to that of controls, we performed PICRUSt analysis, the results of which were visualized with STAMP (Figure 6) [37,38]. The 16S rRNA gene marker sequences of bacteria identified in PKU and control samples along with open-source tools were integrated by PICRUSt2 in order to predict functional potential of the PKU gut microbiome and the control gut microbiome.

Significance (*p* < 0.05) was found in six MetaCyc pathways; four pathways were enriched in the PKU samples (biotin biosynthesis II, superpathway of N-acetylneuraminate degradation, allantoin degradation to glyoxylate III, and gluconeogenesis I) and two pathways were decreased in PKU samples (creatinine degradation II and superpathway of 2,3-butanediol biosynthesis).

## 4. Discussion

In this study, we sequenced the V4 hypervariable region of the 16S rRNA gene from stool samples collected from adult PKU and non-PKU control individuals. In order to understand the effects of PKU on the gut microbiome, we explored the gut microbial community composition, diversity, and predicted changes in metabolic potential in relation to non-PKU controls.

Broadly examining the gut microbial composition at the phylum level, Bacteroidetes and Firmicutes dominate the population in both the PKU and control samples, followed by Actinobacteria, Proteobacteria, and Verrucomicrobia (Figure 1). There is currently no consensus on phylum-level comparisons between previous studies involving children with PKU, and no prior studies have been published on the gut microbiome of adults diagnosed with PKU [23,24]. We then took a genus-level approach to describe relative abundance and identify potential bioindicators of PKU with DESeq2 and Phylofactor, respectively.

DESeq2 analysis found 65 genera were significantly differentially abundant (Figure 2). Of note is *Faecalibacterium*, which was shown here to be decreased in PKU adult samples, and has been reported in previous studies investigating PKU children [23,24]. *Faecalibacterium* consume dietary fiber found in whole foods and produce short chain fatty acids (SCFA), mainly butyrate, which acts as a pH buffer and primary source of energy for colonocytes [40]. These bacteria possess anti-inflammatory properties through production of butyrate and microbial anti-inflammatory molecule (MAM) [41,42,43,44]. Interestingly, Crohn’s disease, IBD, and colitis patients are among several groups found to also contain lower levels of *Faecalibacterium* in stool samples [45,46,47]. As previously mentioned, the PKU diet can be low in protein, whole foods, and fiber. Conversely, the “Western diet” largely consists of processed, high fat, high protein foods, yet those consuming this diet house a gut microbiome also low in the beneficial, fiber-degrading microbes such as *Faecalibacterium* [48]. Future studies may investigate similar properties of these opposing diets to determine the factor responsible for a decreased abundance of beneficial, anti-inflammatory microbes.

Phylofactorization of microbiome data from PKU and control samples revealed three factors to be influential at the genus level (Figure 3): *Romboutsia*, two unknown Lachnospiraceae genera, and *Enterocloster*. Currently, the specific roles of *Romboutsia* and *Enterocloster* are largely unknown due to recent isolations and classifications; however, further studies of the bacteria may expose the importance of their presence [39,49]. Potentially relevant to PKU is that isolated *Romboutsia* genera lack a catalytic enzyme for the biosynthesis of phenylalanine and tyrosine [50]. Alternatively, Lachnospiraceae make up the core human gut microbiome and abundance variations span several diseases and disorders linked to gut dysbiosis, leading to inconsistent conclusions on the role the microbe may play [51]. 

Ecological diversity was determined by several measures of alpha and beta diversity. Alpha diversity was considered through observed ASV counts and calculation of the Shannon diversity index. Previous studies by Bassanini et al. and Pinheiro de Oliveira et al. reported significantly decreased alpha diversity in stool samples from PKU children compared to non-PKU children [23,24]. In this study, we found that while alpha diversity similarly trended lower in PKU adults, the effect was not significant. Further investigation is necessary to determine if the PKU diet and treatment has a more pronounced impact on the gut microbiome of children, which is still developing and not as stable as the adult gut microbiome [52]. Alternatively, differences in microbial diversity between PKU children and adults may be a function of a more regulated diet in children as, typically, parents are planning meals and maintaining a sense of stability, which may lead to better adherence to the diet and subsequently a more dramatic drop in gut microbial diversity. Adults with PKU, on the other hand, may deviate more often from the prescribed diet.

Dysbiosis is often defined by alterations or deviance from “normal” or “healthy” microbiota and is often associated with disease [53]. In order to determine if the PKU gut microbiome profile was significantly deviated from that of non-PKU controls, we performed a PERMANOVA. Results verified a significant difference between the two groups. PKU patients often experience comorbidities spanning multiple organ systems including autoimmune, gastrointestinal, and neuropsychiatric disorders; some of which have been associated with dysbiosis [54,55,56]. Further studies are necessary to determine if the variances described here can be implicated in PKU comorbidities.

As previously mentioned, gut microbiome composition can be shifted through extrinsic mechanisms such as diet, medication, and environment; however, microbiome influence can be bidirectional in that gut microbiota shifts have been shown to contribute to changes in the immune system and even mood and cognition through the gut–brain axis [57,58].

Finally, we found significant differences in predicted metabolic pathways between PKU-positive individuals on a Phe-restrictive diet and control individuals. Pathways that were upregulated in PKU patients included those involved in carbon fixation and carbohydrate fermentation. Interestingly, N-acetylneuraminate degradation was found to be higher in PKU patients; a pathway that has been previously identified as an important factor for pathogenic bacterial proliferation and permeation into the bloodstream in immunocompromised individuals [59]. Identification of pathways that impact the pathogenicity of bacteria may shed light on the higher susceptibility of PKU patients to infectious diseases—including gastroenteritis, colitis, urticaria, rhinitis, and tonsillitis—as compared to the general population [54,60]. Though these imputations are limited, they highlight the need to further explore the effect of the PKU diet on PKU patients from multiple angles, including metabolomics and transcriptomics.

Although the current diet-based treatment is effective at maintaining low Phe levels, the impact of the PKU diet and treatment over the course of a patient’s lifetime remains an unknown. As the gut microbiome can affect distal organs and systems, it is important to fully characterize the impact of this treatment on the gut microbiome in PKU patients.

In this study, we detected significant differences in the abundance of specific bacterial groups and putative metabolic pathways in adult PKU patients as compared to control individuals. Unlike the previous studies of PKU children, we did not find significant differences in diversity of the gut microbiome. The results of this study illustrate the need for further studies with larger samples sizes and representation (i.e., sex and race) to understand the full effects that PKU dietary restrictions and treatments have on patients over time.

## Figures and Tables

**Table 1:** Baseline characteristics of the subjects.

Observations	PKU (*n* = 11)	Control (*n* = 21)
**Sex**		
Female	4	11
Male	6	10
N/R ^1^	1	0
**Average Age** (years)	33 ± 1.98	29 ± 3.07

^1^ N/R: not reported by participant.

## Data Availability

The data presented in this study are openly available in the European Nucleotide Archive PRJEB42748.
